# Cell Division Cycle Associated Genes as Diagnostic and Prognostic Biomarkers in Hepatocellular Carcinoma

**DOI:** 10.3389/fmolb.2021.657161

**Published:** 2021-03-11

**Authors:** Shan-Shan Jiang, Sheng-Jie Ke, Zun-Li Ke, Juan Li, Xiang Li, Xing-Wei Xie

**Affiliations:** ^1^ Key Laboratory of Forensic Toxicology of Herbal Medicines, Guizhou Education Department, School of Basic Medicine, Guizhou University of Traditional Chinese Medicine, Guiyang, China; ^2^ Faculty of Basic Medicine, Zhengzhou Shuqing Medical College, Henan, China; ^3^ College of Plant Protection, Henan Agricultural University, Zhengzhou, China

**Keywords:** CDCA, hepatocellular carcinoma, biomarkers, prognosis, tumor immunity

## Abstract

With high mortality and poor prognosis, hepatocellular carcinoma (LIHC) has become the fourth leading cause of cancer-related deaths worldwide. Most of the LIHC patients missed the best treatment period because of the untimely diagnosis. For others, even if they are temporarily cured, they have to face a very low prognostic survival rate and a very high risk of recurrence. Based on the characteristics of abnormal proliferation and uncontrolled growth of tumor cells. Cell Division Cycle Associated (*CDCA*) family genes, which are responsible for regulating the cell cycle and proliferation, were selected as our research object to explore the mechanism of hepatocarcinogenesis. To this end, we investigated the expression profiles of *CDCA* family genes in LIHC and corresponding normal tissues, and the effect of *CDCAs* expression on the survival of prognosis and immune cell infiltration through bioinformatics analysis methods and the publicly accessible online databases. In addition, we also analyzed the expression correlation of *CDCAs* and screened the neighboring genes related to functional *CDCAs*. The results revealed that the expression levels of *CDCA1/3/5/8* were significantly increased in LIHC, regardless of stage, sex, race, drinking behavior, and other clinical factors. *CDCAs* expression was significantly correlated with poor prognosis and was positively correlated with the infiltration of dendritic cells, B cells, and macrophages. We also found that the most relevant neighboring genes to *CDCAs* in LIHC were *SGO2*, *NDC80*, *BIRC5*, *INCENP*, and *PLOD1*. In general, our work suggests that *CDCA1/3/5/8* has the potential to be a diagnostic gene in hepatocarcinogenesis and prognostic biomarkers for LIHC patients.

## Introduction

Liver cancer is the fourth leading cause of cancer-related deaths worldwide, with an estimated incidence rate of over one million cases per year, which seriously endangers human health (Bray et al., 2018; Llovet et al., 2018). Hepatocellular carcinoma (LIHC), accounting for 75–85% of cases, is the main pathological type of primary liver cancer (Bray et al., 2018). The majority of LIHC cases arise from hepatitis B and hepatitis C in hepatocytes (Zhang et al., 2004; Makarova et al., 2016). Additionally, the rising incidence of LIHC is due to a high rate of alcohol consumption and the occurrence of non-alcoholic fatty liver disease (Schütte et al., 2009; Gish et al., 2016). Although the development of various treatment technologies, including surgical resection, liver transplantation surgery, interventional therapy, chemotherapy, and radiotherapy, may reduce mortality to some extent, LIHC patients still bear a poor five-year survival rate of only 20–30% (Pang et al., 2008; Yang et al., 2009). Thus, there is an urgent need to deepen the understanding of LIHC tumorigenesis and develop new treatment and monitoring methods for early detection and prolong the survival of LIHC patients.

Dysregulation in any process of cell division may lead to malignancy (Collins and Garrett, 2005). Cell Division Cycle Associated (*CDCA*) family proteins, including eight respective members of *CDCA1-8*, function to regulate the cell cycle and proliferation, which play an important role in biological process. Previous studies have shown that the expression levels of all or part of *CDCA* family genes are significantly upregulated in lung cancer, breast cancer, renal cell cancer, etc. This high expression is usually associated with a poor prognosis. For example, *CDCA* genes are highly expressed in ovarian cancer tissues and act on the PLK pathway to promote tumor invasion and metastasis (Chen et al., 2020a; Chen et al., 2020b). *CDCA7* can accelerate the proliferation of lung adenocarcinoma and non-small cell lung cancer by regulating the cell cycle (Wang et al., 2019). High mRNA expression of *CDCA3/5/7/8* in breast cancer significantly reduced the survival rate of patients (Phan et al., 2018). These findings strongly suggest the potential role of *CDCAs* in the tumorigenesis and prognosis of patients. It is necessary and meaningful to understand the mechanism of action of *CDCAs* in LIHC. To date, however, there is no systematic and comprehensive analysis of *CDCAs* in LIHC.

To reveal the mechanism of LIHC tumorigenesis and to identify diagnostic and prognostic markers or therapeutic targets for LIHC patients, in the current study, the transcriptional levels of *CDCA* genes were investigated by Oncomine and GEPIA, and we found that *CDCA1/3/5/8* expression levels were significantly increased in LIHC. We also evaluated the impact of *CDCA1/3/5/8* on the prognosis of LIHC patients. Specifically, we analyzed the effect of *CDCAs* expression on the prognostic survival rate of LIHC patients, the correlation between *CDCAs* transcription and various clinical factors by Kaplan-Meier plotter, and the effect of genetic alterations of *CDCAs* on prognosis by cBioPortal. The relationship between *CDCA1/3/5/8* and immune cell infiltration was analyzed using TIMER 2.0. In addition, the interaction network between *CDCAs* and neighboring genes was mapped using GeneMANIA.

## Materials and Methods

### Oncomine Analysis

The Oncomine platform (https://www.oncomine.org/) is a publicly accessible online tumor-related gene microarray database that collects related gene expression profiles and relevant clinical information. The transcriptional levels of *CDCA* family genes (*CDCA1–8*) in different tumors and corresponding normal tissues were analyzed by Oncomine with approximately 200 samples. The expression levels were considered significantly different when fold change > 2.0 and *p*-value < 0.0001. We set the threshold value of gene rank to “top 10%” and the data type to “mRNA” (Rhodes et al., 2007).

### GEPIA Analysis

GEPIA (http://gepia.cancer-pku.cn/index.html) was used to analyze the expression of *CDCAs* sequencing in liver cancer tissue based on the GTEx and TCGA databases (Tang et al., 2017). GEPIA was used to compare the *CDCAs* expression levels in HCC with the thresholds of |Log2 (Fold Change)| Cut-off: 1 and *p*-value Cut-off: 0.01. *CDCAs* expression in different HCC stages was analyzed using the default parameters.

### Kaplan-Meier Plotter Analysis

Kaplan-Meier plotter (http://kmplot.com/analysis/) was used to evaluate the influence of different expression levels of *CDCA* family genes on prognostic value, including overall survival (OS), disease-specific survival (DSS), relapse-free survival (RFS), progression-free survival (PFS), and OS of liver cancer patients with different clinical factors. We analyzed all samples in the database with the parameters of Group Cut-off: Median; Hazards Ratio: Yes; 95% Confidence Interval: Yes; Follow-up threshold: All.

### cBioPortal Analysis

cBioPortal (http://www.cbioportal.org/) was used to perform the interactive analysis of biomolecules in tumor tissues in the TCGA database (Gao et al., 2016). Here, we used it to analyze the alterations in the frequency of *CDCAs* genes change. Putative copy-number calls on 370 cases determined using GISTIC 2.0. In module Comparison/Survival, we analyzed the influence of alterations on prognostic survival in HCC patients by default parameters.

### GeneMANIA Analysis

GeneMANIA (http://genemania.org/), based on many large, publicly available biological datasets, is used to identify intra-genomic associations and find co-expressing biomolecules. Here, GeneMANIA was used to identify the physical interactions and co-expression of *CDCAs* with 20 related genes in Homo sapiens datasets by default parameters.

### TIMER 2.0 Analysis

Using TIMER 2.0 (http://timer.comp-genomics.org/), we analyzed the relationship between the expression of *CDCAs* and infiltration levels of immune cells in liver cancer tissue. The TIMER database was used to determine the abundance of tumor infiltrates based on biomarker gene expression analysis (Li et al., 2017). Here, we chose *CDCA1/3/5/8* as input and, in turn, detected cancer cells under the Immune Association module. B cells, CD8+ T cells, CD4+ T cells, neutrophils, macrophages, and dendritic cells were selected as the test types according to Li et al. (Li et al., 2016; Danaher et al., 2017). Gene expression values were transformed to Log2 RSEM values.

## Results

### Expression of Cell Division Cycle Associateds in Hepatocellular Carcinoma and Other Cancers

**FIGURE 1:**
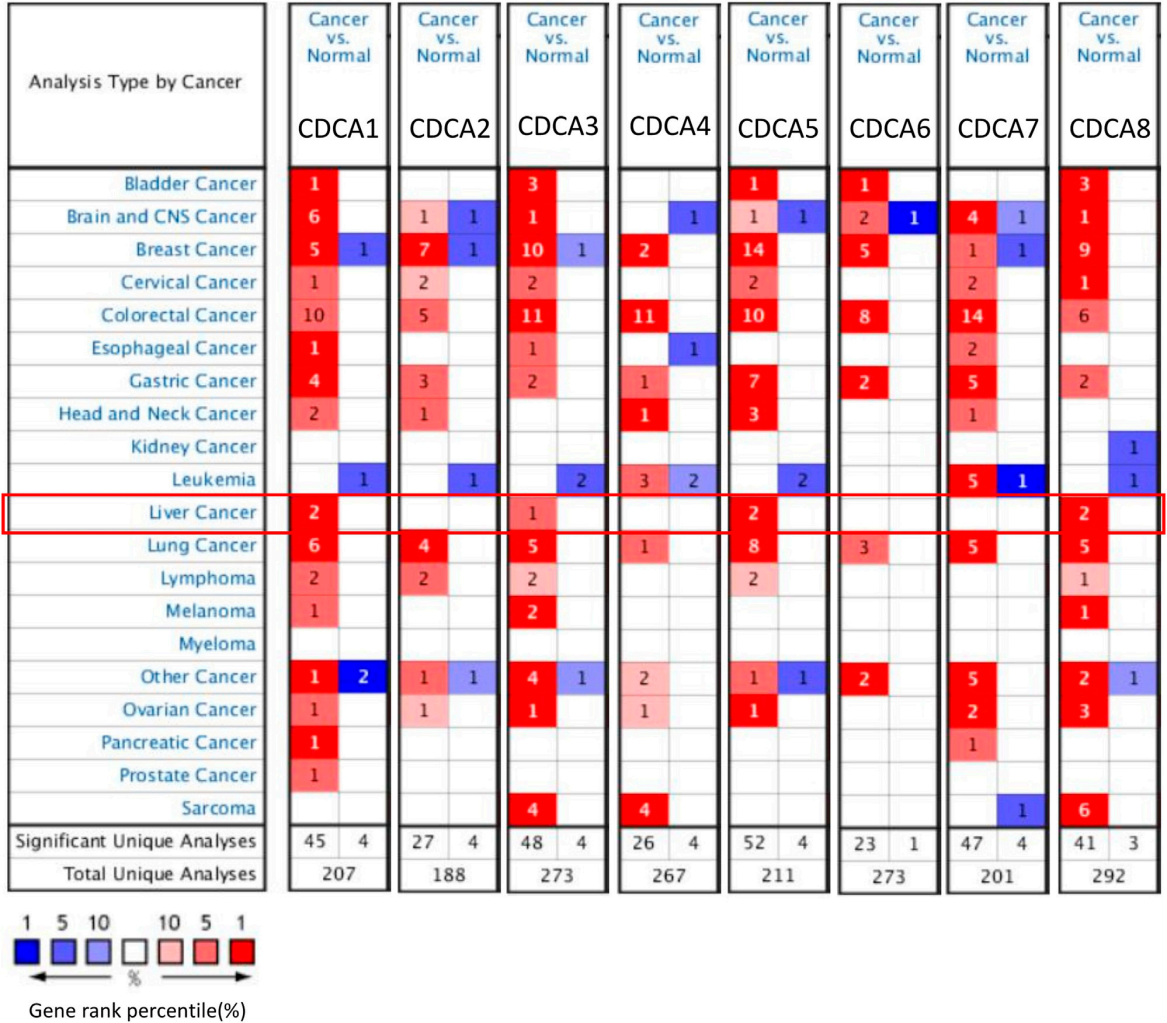
Expression levels of *CDCAs* in various normal or cancer tissues via Oncomine analysis.

**FIGURE 2:**
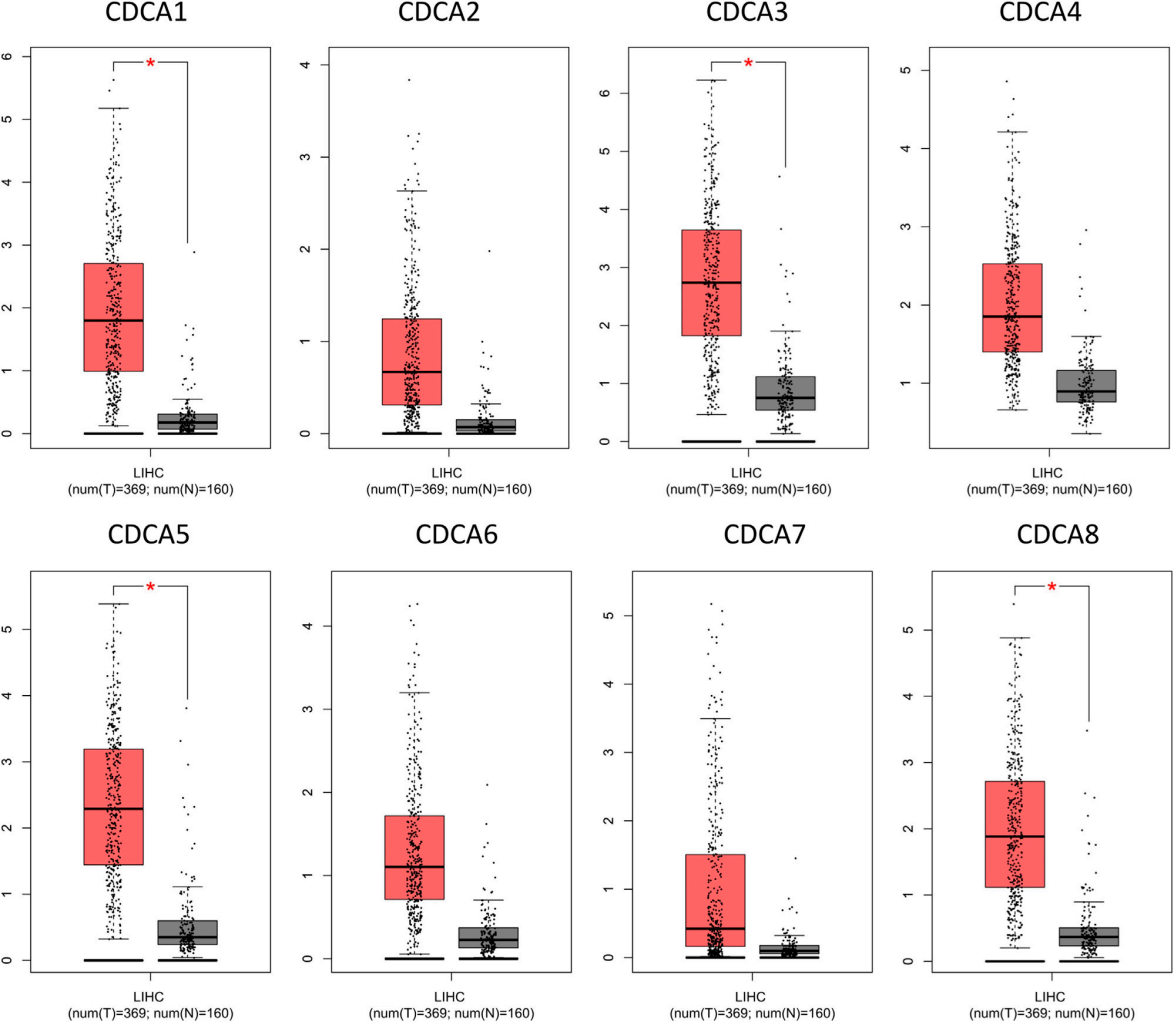
Expression levels of *CDCAs* in LIHC via GEPIA analysis.

Dysregulation in any process of cell division may lead to malignancy. It has been reported that there are eight respective members in the *CDCA* family genes, among which members may play an independent role or function cooperatively. Oncomine analysis revealed that most *CDCA* gene members were significantly upregulated in 15 cancer types (Figure 1). For LIHC, in particular, we found consistent results in both the Oncomine and GEPIA databases, i.e., the expression of *CDCA1*, *CDCA3*, *CDCA5*, and *CDCA8* in LIHC was significantly higher than those in normal tissues (Figures 1, 2A). Therefore, these genes were selected for follow-up analysis and research objects.

Tumor development is usually subdivided into four stages to assess the extent of cancer cell proliferation and to determine the prognostic potential of patients. In this study, significant differences were observed in the above four members of *CDCA* genes in different stages of liver cancer, suggesting that these four genes may function in the whole course of LIHC tumorigenesis (Figure 2B).

### Prognostic Potential of Cell Division Cycle Associateds Expression in Hepatocellular Carcinoma

**FIGURE 3:**
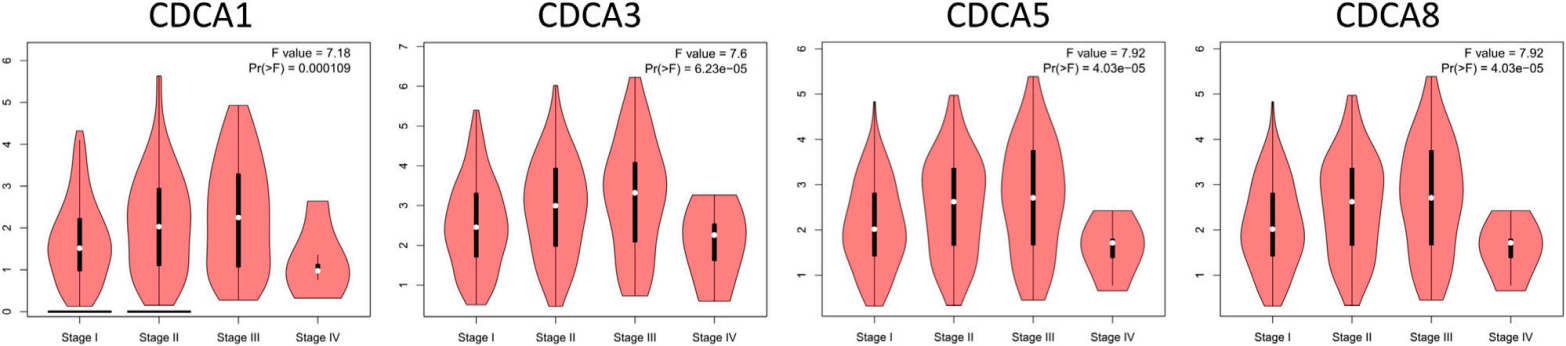
Correlation of *CDCAs* expression with tumor stage among LIHC cases via GEPIA analysis.

**FIGURE 4:**
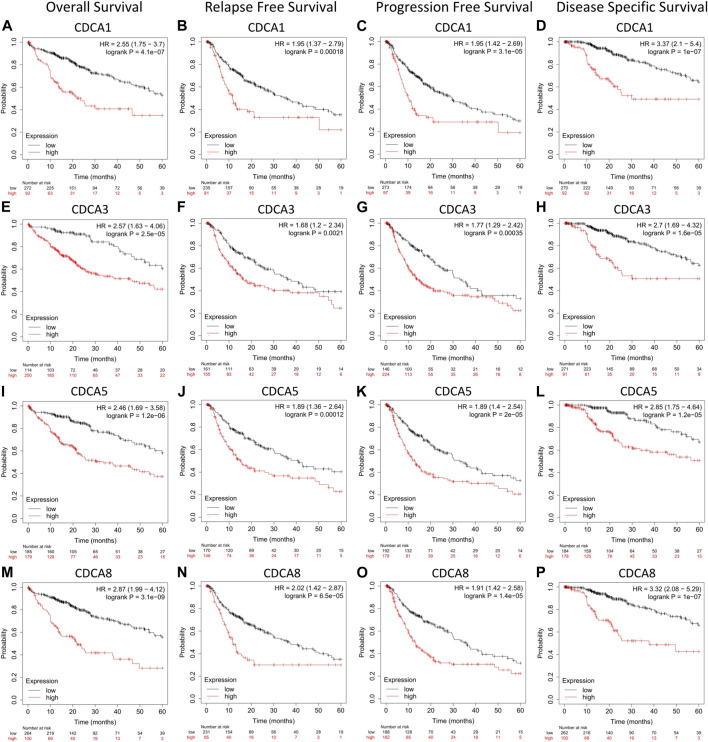
Correlation analysis between *CDCAs* expression and prognostic survival in LIHC patients via Kaplan-Meier plotter analysis. **(A‐D)** Correlation between expression of *CDCA1* and survival curves. **(E‐H)** Correlation between expression of *CDCA3* and survival curves. **(I‐L)** Correlation between expression of *CDCA5* and survival curves. **(M‐P)** Correlation between expression of *CDCA8* and survival curves.

Using the Kaplan-Meier plotter, we found that the transcription level of the four test *CDCA* genes was significantly correlated with the prognostic survival rate of liver cancer patients (Figure 3). Specifically, high expression of *CDCAs* in OS, DSS, RFS, and PFS represented poor prognosis, indicating that active transcription of *CDCA1/3/5/8* might cause health risks, and these genes could be potential prognostic biomarkers for LIHC patients (Figure 4).

For different types of clinical factors, we further analyzed the relationship between the expression levels of *CDCA1/3/5/8* genes and prognostic OS (Table 1). Here, we concluded that the high expression levels of *CDCAs* led to poor prognosis regardless of sex, race, or alcohol consumption. Notably, when there was no hepatitis virus or vascular infection in LIHC patients, the expression of *CDCA1/3/5/8* was significantly correlated with the survival rate. Interestingly, only *CDCA8* was significantly associated with survival in patients with hepatitis virus or vascular infection (Table 1).

**TABLE 1:** Correlation analysis between *CDCAs* expression and prognostic overall survival in LIHC patients with different clinicopathological factors via Kaplan-Meier plotter analysis.

Clinical factors			CDCA1	CDCA3	CDCA5	CDCA8
**Gender**	male	HR (95% CI)	3.90	2.91	2.67	2.72
		*p*-value	**1.60E-05**	**1.60E-04**	**2.30E-05**	**9.50E-06**
	female	HR (95% CI)	2.37	2.73	2.16	3.16
		*p*-value	**5.30E-03**	**6.30E-04**	**0.012**	**7.50E-05**
**Race**	White	HR (95% CI)	2.42	3.30	1.70	2.31
		*p*-value	**1.90E-03**	**1.60E-03**	**0.029**	**5.30E-03**
	Asian	HR (95% CI)	5.63	4.14	6.26	6.19
		*p*-value	**2.40E-09**	**5.70E-07**	**8.80E-08**	**1.20E-10**
**Alcohol consumption**	yes	HR (95% CI)	5.26	3.03	3.39	4.12
		*p*-value	**1.80E-04**	**6.60E-03**	**4.10E-03**	**7.60E-04**
	none	HR (95% CI)	2.70	2.21	2.45	3.42
		*p*-value	**3.70E-05**	**8.90E-04**	**3.30E-04**	**1.10E-07**
**Hepatitis virus**	Yes	HR (95% CI)	1.94	1.68	1.93	2.26
		*p*-value	**0.04**	0.13	0.05	**0.02**
	None	HR (95% CI)	3.05	3.76	3.39	4.09
		*p*-value	**4.50E-06**	**5.20E-06**	**4.40E-06**	**4.80E-06**
**Vascular invasion**	yes	HR (95% CI)	2.65	2.63	2.37	4.54
		*p*-value	0.11	0.09	0.09	**0.04**
	none	HR (95% CI)	3.66	6.13	5.64	0.06
		*p*-value	**0.01**	**5.70E-03**	**0.0098**	**5.70E-03**

Bold values indicate *p* < 0.05.

### Alteration Analysis of Cell Division Cycle Associateds in Hepatocellular Carcinoma

**FIGURE 5:**
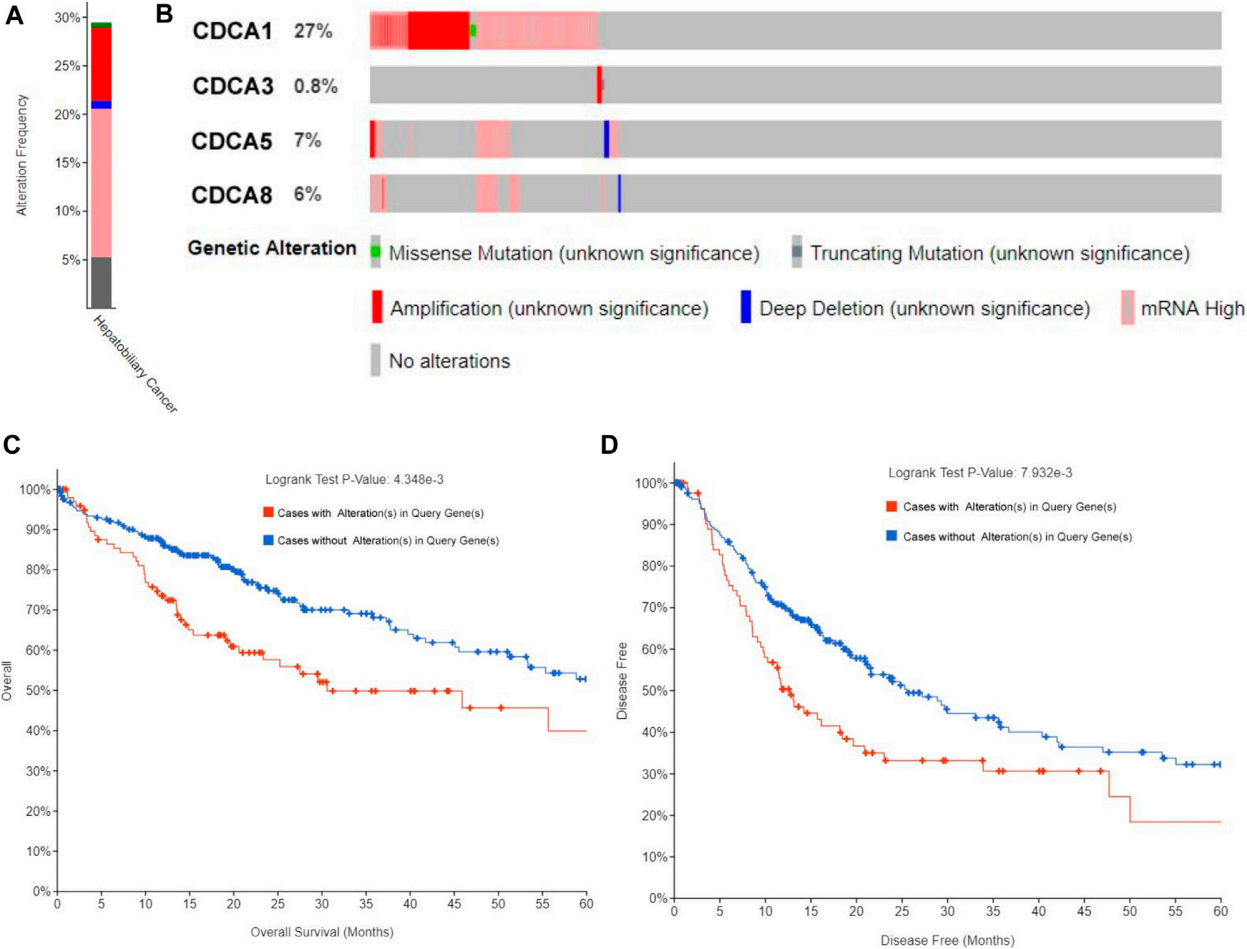
Alteration frequency analysis of *CDCAs* and its influence on prognosis in LIHC patients via cBioPortal analysis. **(A,B)** Alteration frequency of *CDCAs* based on the TCGA provisional dataset. **(C,D)** Kaplan-Meier plots comparing OS **(C)** and DFS **(D)** in cases with/without *CDCAs* genetic alterations.

We extracted *CDCA1/3/5/8* genes and investigated the percentages of genetic alterations in the TCGA dataset. In a total of 360 samples, the alteration frequency of *CDCAs* was as high as 29.44% in LIHC (106/360) (Figure 5A), with 27% of *CDCA1*, 0.8% of *CDCA3*, 7% of *CDCA5*, and 6% of *CDCA8* (Figure 5B). Next, we performed a correlation analysis between cases with (or without) *CDCAs* genetic alterations and prognostic survival. There was a significant correlation between *CDCA* alterations and survival of both OS (4.348e-3) (Figure 5C) and DFS (7.932e-3) (Figure 5D), implying that these alterations will aggravate the hepatocarcinogenesis mediated by high expression of *CDCAs*, which is not conducive to the survival of patients.

### Correlation Analysis Between Cell Division Cycle Associateds Expression Levels and Immune Cell Infiltration in Hepatocellular Carcinoma

**FIGURE 6:**
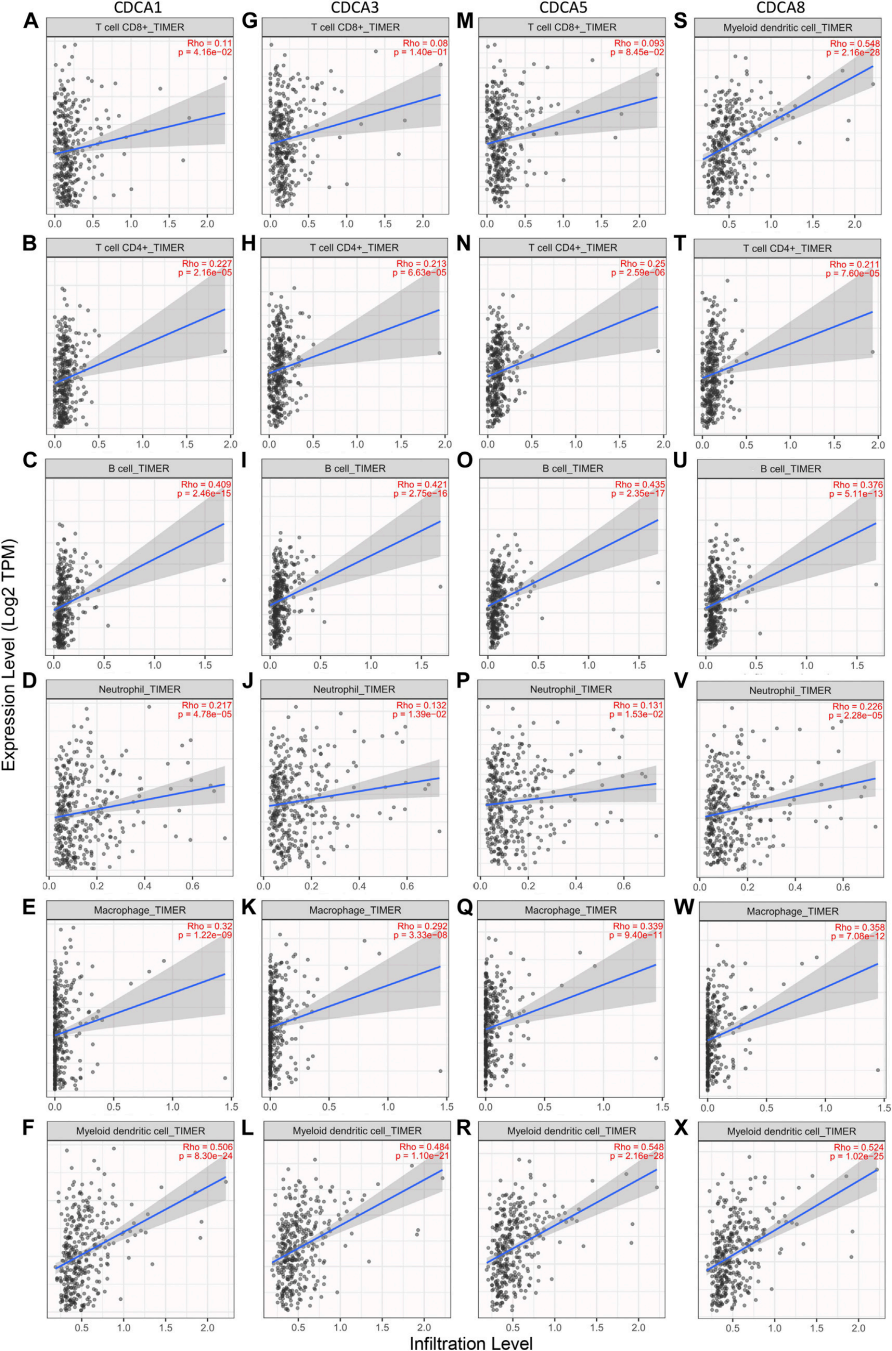
Correlation analysis between *CDCAs* expression and immune cell infiltration levels in LIHC patients via TIMER 2.0 analysis. **(A‐F)** Correlation analysis between *CDCA1* expression and immune cell infiltration levels. **(G‐L)** Correlation analysis between *CDCA3* expression and immune cell infiltration levels. **(M‐R)** Correlation analysis between *CDCA5* expression and immune cell infiltration levels. **(S‐X)** Correlation analysis between *CDCA8* expression and immune cell infiltration levels.

TIMER 2.0 was used to investigate the correlation between the expression of *CDCA1/3/5/8* and infiltration levels of immune cells (CD8+ T cells, CD4+ T cells, B cells, neutrophils, macrophages, and dendritic cells). Overall, there was a statistically significant positive correlation between *CDCAs* gene expression and most immune cell infiltration in LIHC. In detail, *CDCAs* showed a poor correlation with CD8+ T cells and neutrophils, but a stronger correlation with dendritic cells, B cells, and macrophages (Figure 6).

To provide a basis for exploring the immune mechanisms mediated by *CDCAs* and screening potential therapeutic targets, we further investigated the correlation of expression levels between *CDCA1/3/5/8* and biomarker genes of immune cells and their subsets in LIHC. We found that the *CDCA* gene was positively correlated with all test biomarker genes of dendritic cells and B cells (Table 2). Although *CDCAs* showed a significant positive correlation with most of the biomarker genes of M1 macrophage and M2 macrophage, there was no correlation between *CDCA1/3/5/8* and *iNOS* (macrophage biomarker). In addition, there was no association between *CDCA3* and *COX2* or *CD163* (Table 2).

**TABLE 2:** Correlation analysis between the expression of *CDCAs* and immune cell biomarker genes in LIHC patients via TIMER 2.0 analysis.

Description	Gene markers	CDCA1		CDCA3		CDCA5		CDCA8	
		Rho	*p* -value	Rho	*p* -value	Rho	*p* -value	Rho	*p* -value
**B Cell**	CD19	0.372	**8.8E-13**	0.319	**1.38E-09**	0.315	**2.13E-09**	0.320	**1.19E-09**
	CD79A	0.291	**3.54E-08**	0.266	**5.19E-07**	0.260	**9.59E-07**	0.233	**1.19E-05**
**M1 Macrophage**	NOS2	0.028	6.09E-02	-0.061	2.26E-01	-0.018	7.34E-01	0.052	3.33E-01
	IRF5	0.437	**1.64E-17**	0.372	**9.36E-13**	0.379	**3.15E-13**	0.425	**1.33E-16**
	PTGS2	0.207	**1.09E-04**	0.089	1.00E-01	0.167	**1.83E-03**	0.218	**4.39E-05**
**M2 Macrophage**	CD163	0.136	**1.14E-02**	0.077	1.54E-01	0.159	**3.13E-03**	0.194	**2.99E-04**
	VSIG4	0.17	**1.58E-03**	0.117	**3.03E-02**	0.196	**2.40E-04**	0.230	**1.65E-05**
	MS4A4A	0.194	**2.82E-04**	0.236	**9.04E-06**	0.207	**1.04E-04**	0.234	**1.16E-05**
**Dendritic cell**	HLA-DPB1	0.258	**1.22E-06**	0.25	**2.61E-06**	0.297	**1.77E-08**	0.277	**1.78E-07**
	HLA-DQB1	0.231	**1.53E-05**	0.245	**3.96E-06**	0.28	**1.29E-07**	0.253	**1.92E-06**
	HLA-DRA	0.295	**2.47E-08**	0.236	**9.26E-06**	0.287	**5.95E-08**	0.309	**4.35E-09**
	HLA-DPA1	0.256	**1.41E-06**	0.203	**1.47E-04**	0.264	**6.75E-07**	0.290	**4.35E-08**
	CD1C	0.202	**1.59E-04**	0.159	**3.00E-02**	0.187	**4.88E-04**	0.171	**1.46E-03**
	NRP1	0.224	**2.73E-05**	0.141	**8.74E-03**	0.177	**9.40E-04**	0.274	**2.45E-07**
	ITGAX	0.442	**6.51E-18**	0.408	**2.98E-15**	0.445	**3.51E-18**	0.467	**4.50E-20**

Bold values indicate *p* < 0.05.

### Co-Expression and Interaction of Cell Division Cycle Associateds in Hepatocellular Carcinoma

**FIGURE 7:**
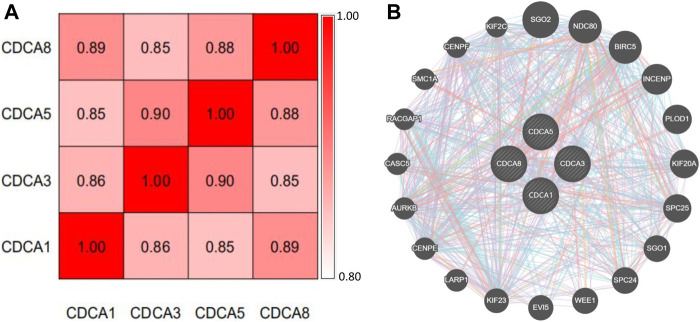
Co-expression and interaction of *CDCAs* at the gene and protein levels in LIHC patients. **(A)** Spearman’s correlation analysis of *CDCAs*. **(B)** Gene–gene interaction network among *CDCAs* in the GeneMANIA dataset.

At the genetic level, the expression correlation of the four member genes of *CDCA1/3/5/8* in liver cancer patients was analyzed using cBioPortal. According to our results, the Pearson correlation coefficients ranged from 0.85–0.90, indicating a strong correlation between the expression patterns of these four *CDCAs* in LIHC (Figure 7A). The GeneMANIA dataset was used to explore the co-expression of *CDCAs* with other biomolecules. Here, we found that there are physical interactions between *CDCA1*, *CDCA3*, *CDCA5*, and *CDCA8*. The interaction network of CDCAs and the 20 most frequently neighboring genes are shown in Figure 7B. The top five *CDCAs* neighboring genes in LIHC were *SGO2*, *NDC80*, *BIRC5*, *INCENP*, and *PLOD1*.

## Discussion

With high mortality and poor prognosis, LIHC has become the fourth leading cause of cancer-related deaths worldwide (Bray et al., 2018). Most of the LIHC patients missed the best treatment period because of the untimely diagnosis. For others, even if they are temporarily cured, they have to face a very low prognostic survival rate and a very high risk of recurrence. Based on the characteristics of abnormal proliferation and uncontrolled growth of tumor cells. *CDCA* genes, which are responsible for regulating the cell cycle and proliferation, were selected as our research object to explore the mechanism of hepatocarcinogenesis. Through a series of bioinformatics analysis methods and the use of publicly accessible online databases, including Oncomine, Kaplan-Meier plotter, GEPIA, GeneMANIA, cBioPortal, and TIMER 2.0, we investigated the expression profiles of *CDCA* family genes in LIHC and corresponding normal tissues, and the effect of *CDCAs* expression on the survival of prognosis and immune cell infiltration. In addition, we also analyzed the expression correlation of *CDCAs* and screened the neighboring genes related to functional *CDCAs*.

We found that the expression levels of *CDCA1/3/5/8* were significantly increased in LIHC, regardless of stage, sex, race, drinking behavior, and other clinical factors. *CDCAs* expression was significantly correlated with poor prognosis of OS, DSS, RFS, and PFS and was positively correlated with the infiltration of dendritic cells, B cells, and macrophages. We also found that the most relevant neighboring genes to *CDCAs* in LIHC were *SGO2*, *NDC80*, *BIRC5*, *INCENP*, and *PLOD1*. In general, our work suggests that *CDCA1/3/5/8* has the potential to be a diagnostic gene in hepatocarcinogenesis and prognostic biomarkers for LIHC patients.

The *CDCA* family consists of eight independent individuals named *CDCA1–8*. To clarify the role of *CDCAs* in LIHC, multiple database analysis was performed, and *CDCA1/3/5/8* was found to be highly expressed in LIHC tissue. Moreover, this active expression of *CDCAs* was observed in all stages of LIHC, indicating that *CDCA1/3/5/8* has potential as a diagnostic gene for the occurrence and development of LIHC. Similarly, *CDCA* has been shown to be highly expressed in many other cancer types, such as breast cancer and lung cancer, indicating its wide applicability and functional conservation. However, there was no significant upregulation of *CDCAs* in leukemia, suggesting that it is necessary to distinguish the types of cancer when they were used as diagnostic genes.

According to previous reports, *CDCA1*, also known as *Nuf2*, is mainly responsible for regulating cell mitosis (Zhang et al., 2015). Downregulation of *CDCA1* expression can inhibit the proliferation of tumor cells, while overexpression of *CDCA1* is associated with poor prognosis (Hu et al., 2015). *CDCA3* has been shown to promote cell proliferation and invasion through activation of the Ras signaling pathway or hypomethylation in gastric cancer cells (Zhang et al., 2019). *CDCA5* plays important roles in migration, proliferation, apoptosis, and invasion of tumor cells by regulating sister chromatid segregation and cohesion (Xu et al., 2019). *CDCA8* is an integral part of the chromosomal passenger complex, which is involved in mitosis and contributes to distant metastasis of cancer cells (Jeon et al., 2017). It is worth noting that the high expression of these four genes has been proven to reduce the prognostic survival rate of many cancer patients. This is consistent with the results of our study. In particular, there is a significant negative correlation between *CDCAs* and four common prognostic indices of OS, DSS, RFS, and PFS, and this correlation is applicable to different clinical factors, suggesting that high expression of *CDCAs* may be one of the causes of poor prognosis. These biomolecules have the potential to be prognostic biomarkers for LIHC patients.

However, in the presence of pathological conditions, such as hepatitis virus or vascular invasion, there was no correlation between *CDCAs* and OS, except for *CDCA8*, suggesting that we should consider the patient’s clinical condition when selecting the diagnostic gene. In addition, we found that the alteration frequency of *CDCAs* is extremely high, and the genetic alteration will reduce the prognosis survival of LIHC patients. The factors causing gene alteration should be avoided in the process of treatment, and the degree of an alteration should be properly considered when detecting expression and evaluating prognosis.

Dendritic cells are special antigen-presenting cells that play a major role in activating T lymphocytes with anti-tumor effects (Wculek et al., 2020). B cells can produce a kind of IgG antibody to recognize a certain antigen in tumor tissue, which can inhibit tumor growth (Li et al., 2009). Macrophages were divided into M1 macrophages and M2 macrophages. M1 macrophages are mainly related to the recognition and attack of tumor cells, while M2 macrophages are related to tumor progression and immunosuppression (Yang and Zhang, 2017; Shapouri et al., 2018). *CDCAs* showed a significant positive correlation with dendritic cells, B cells, and macrophages, indicating that *CDCA*-mediated hepatocarcinogenesis might mobilize the activity of these immune cells and make them play an anti-tumor role. When we further analyzed the relationship between *CDCA1/3/5/8* expression and the biomarker genes of immune cells, we found a significant positive correlation between *CDCAs* expression and all test biomarkers of B cells and dendritic cells. However, for M1 macrophages and M2 macrophages, *CDCAs* were only related to some of macrophages biomarker genes, indicating that there is a certain specificity and selectivity in this interaction, which also provides some basis for immunotherapy in the future.

Although these four genes have some differences in function and target genes (pathways), they jointly regulate the cell division cycle to promote the proliferation and invasion of tumor cells. The Pearson correlation coefficients of these four genes were all greater than 0.85. GeneMANIA analysis showed twenty neighboring genes to *CDCAs*, and the functions of these genes are also related to cell cycle regulation. For example, *SGO1* is considered to play a major role in recruiting the chromosomal passenger complex to chromosomes (Bonner et al., 2020); *SGO2* is a pericentromeric protein that associates with cohesin at centromeres and regulates chromosomal segregation during meiosis (El et al., 2017). *SGO1/2* functions as an essential protector for centromeric cohesion and is required for accurate chromosome segregation during mitosis and meiosis (Dudas et al., 2011). *PLOD1* has been reported to function in extracellular matrix formation and is involved in various diseases, including cancer (Wang et al., 2018). The *NDC80* complex, including the main elements of *NDC80*, *SPC24*, and *SPC25*, is highly expressed in various tumors and cooperatively promotes the invasion and metastasis of tumor cells (Alushin et al., 2012; Umbreit et al., 2012). *BIRC5* is a negative regulatory protein that inhibits tumor cell apoptosis and promotes cell proliferation (Cho et al., 2020; Shi et al., 2020).

These proteins are more or less involved in the development of tumors, which explains the mechanism of *CDCA*-mediated hepatocarcinogenesis to a certain extent. This also screened out some targets for future research on new therapeutic methods. However, the specific roles of *CDCAs* in tumorigenesis and development, as well as their interaction with target proteins need to be further verified. The same shortcomings run through our research because the results were based on big data mining and analysis, which inevitably led to some false-positive results. An important purpose was to provide researchers with a more instructive research idea and tried to make our findings point out a direction for future research. Although we mined the data as comprehensively as possible and had obtained some meaningful conclusions, the relevant results still require further experimental and clinical verification.

## Data Availability

The original contributions presented in the study are included in the article/Supplementary Material, further inquiries can be directed to the corresponding authors.
